# White Matter Hyperintensity in Athletes: A cross‐sectional study

**DOI:** 10.1002/alz70857_102283

**Published:** 2025-12-24

**Authors:** Yasmin Soliman, Carmela Tartaglia, Juan‐Camilo Vargas‐González, Charles Tator, Robin Green, Chloe Anastassiadis, Vishaal Sumra, Artee Srivastava, University Health Network

**Affiliations:** ^1^ UHN, Toronto, ON, Canada; ^2^ Canadian Concussion Centre, Krembil Brain Institute, University Health Network, Toronto, ON, Canada; ^3^ University of Toronto, Toronto, ON, Canada; ^4^ Memory Clinic, Toronto Western Hospital, University Health Network, Toronto, ON, Canada; ^5^ Memory Clinic, Toronto Western Hospital, Memory Clinic, Toronto Western Hospital, University Health Network, Toronto, ON, Canada; ^6^ University Health Network, Toronto, ON, Canada; ^7^ Toronto Dementia Research Alliance, Toronto, ON, Canada; ^8^ Canadian Concussion Centre, Toronto Western Hospital, University Health Network, Toronto, ON, Canada; ^9^ Canadian Concussion Centre, Toronto Western Hospital, Krembil Brain Institute, University Health Network, Toronto, ON, Canada; ^10^ Division of Neurosurgery, Toronto Western Hospital, Krembil Brain Institute, University Health Network, Toronto, ON, Canada; ^11^ Institute of Medical Science, University of Toronto, Toronto, ON, Canada; ^12^ KITE Research Institute, University Health Network, Toronto, ON, Canada; ^13^ Tanz Centre for Research in Neurodegenerative Diseases, University of Toronto, Toronto, ON, Canada; ^14^ Krembil Brain Institute, University Health Network (UHN), Toronto, ON, Canada; ^15^ University of Toronto/ University Health Network, Toronto, ON, Canada

## Abstract

**Background:**

Repetitive head impacts (RHI) in athletes, especially those involved in contact sports, have been associated with white matter hyperintensities (WMH), markers of small vessel disease, and neurodegeneration ^1, 2, 3, 4^. This study investigates the relationships between concussion history, vascular risk factors, and WMH volume, as well as their associations with cognitive outcomes in athletes with RHI.

**Method:**

This cross‐sectional study included 96 male professional contact sports athletes enrolled in the Canadian Concussion Centre's concussion monitoring program and 21 male controls without a history of concussion. WMH volumes were quantified from T2‐weighted FLAIR magnetic resonance imaging using automated segmentation. Vascular risk factors were assessed via questionnaires. Cognitive function was evaluated with standardized neuropsychological tests, and composite scores for memory and executive function were derived using normative data for both athletes and controls. All analyses controlled for age. Linear regression models evaluated associations between concussion history (treated as a continuous variable), WMH volume, cognitive performance, and vascular risk factors.

**Result:**

Athletes had significantly greater WMH volume than controls (median 1.3 ml vs. 0.8 ml, *p* =  0.024), although the difference was insignificant when normalized to total intracranial volume. Age was the strongest predictor of WMH volume (β = 0.3851, *p* < 0.001) and was associated with reduced hippocampal (right: β = ‐0.012, *p* < 0.001; left: β = ‐0.011, *p* =  0.001) and amygdala volumes (right: β = ‐0.003, *p* =  0.003; left: β = ‐0.004, *p* =  0.003). Concussion history was associated with declines in executive function (β = ‐0.0244, *p* =  0.034) and memory (β = ‐0.0539, *p* =  0.027) but not with WMH volume. Vascular risk factors were not significantly associated with WMH or cognitive outcomes.

**Conclusion:**

Athletes with RHI demonstrated higher WMH volume than controls, mainly driven by age rather than concussion history or vascular risk factors. Concussion history was associated with cognitive impairments in executive function and memory, emphasizing the importance of cognitive monitoring in aging athletes and the need for longitudinal studies to explore the interplay between RHI, aging, cognition, and neurodegeneration.

